# Effects of electroacupuncture on recovery of the electrophysiological properties of the rabbit gastrocnemius after contusion: an in vivo animal study

**DOI:** 10.1186/s12906-015-0601-z

**Published:** 2015-03-19

**Authors:** Shouyao Liu, Rongguo Wang, Dan Luo, Qianwei Xu, Cheng Xiao, Peng Lin, Zhange Yu, Xuanji Zhao, Rongrong Cai, Jinhui Ma, Qingxi Zhang, Yunting Wang

**Affiliations:** Department of Graduate School, Beijing University of Chinese Medicine, No. 11, North 3rd Ring East Road, Chaoyang District, Beijing 100029 China; Department of Orthopedics, China-Japan Friendship Hospital, No. 2, Yinghua East Road, Chaoyang District, Beijing 100029 China; College of Acupuncture-Moxibustion and Tuina, Beijing University of Chinese Medicine, No. 11, North 3rd Ring East Road, Chaoyang District, Beijing 100029 China; Institute of Clinical Medicine, China-Japan Friendship Hospital, No. 2, Yinghua East Road, Chaoyang District, Beijing 100029 China; Department of Graduate School, Peking University of Health Science Center, No. 38, College Road, Haidian District, Beijing 100191 China

**Keywords:** Electroacupuncture, Electromyography, Nerve conduction velocity, Acetylcholinesterase, Agrin

## Abstract

**Background:**

Our preliminary studies indicated that electroacupuncture (EA) at the ST36 and Ashi acupoints could promote regeneration of the rabbit gastrocnemius (GM) by improving microcirculation perfusion, promoting the recovery of myofiber structures, and inhibiting excessive fibrosis. However, the effects of EA on recovery of the electrophysiological properties of the GM after contusion are not yet clear. Thus, the purpose of this study was to investigate the effects of EA at the Zusanli (ST36) and Ashi acupoints with regard to recovery of the electrophysiological properties of the rabbit GM after contusion.

**Methods:**

Forty-five rabbits were randomly divided into three groups: normal, contusion, and EA. After an acute GM contusion was produced (in rabbits in the contusion and EA groups), rabbits in the EA group were treated with electrostimulation at the ST36 and Ashi acupoints with 0.4 mA (2 Hz) for 15 min. The contusion group received no EA treatment. At different time points (7, 14, and 28 days) after contusion, we performed surface electromyography (EMG) and measured the nerve conduction velocity (NCV) of the GM and the GM branch of the tibial nerve. We also examined acetylcholinesterase (AchE) and Agrin expression in the neuromuscular junction (NMJ) via immunohistochemistry.

**Results:**

Compared with the contusion group, the EMG amplitude and NCV in rabbits in the EA group were significantly higher at all time points after contusion. AchE and Agrin expression in the EA group were significantly higher than those in the contusion group.

**Conclusions:**

Our results showed that EA at the ST36 and Ashi acupoints effectively promoted recovery of the electrophysiological properties of the rabbit GM after contusion. The effects of EA were realized by promotion of the regeneration of myofibers and nerve fibers, as well as acceleration of NMJ reconstruction by upregulation of AchE and Agrin expression in the motor endplate area.

## Background

Skeletal muscle injuries are very common and often occur as unexpected injuries [1]. After a skeletal muscle is injured, regeneration and healing involves repair of the myofiber structure of the muscle, innervation of the nerves and vessels that nourish the muscle, and return of muscle function. The physiological indexes for evaluating skeletal muscle function primarily include the electrophysiological characteristics of the motor unit [2] and biomechanical properties of the muscle [3]. Only skeletal muscle restores its inherent electrophysiological and biomechanical properties, which is the true definition of complete recovery. Surface electromyography (EMG) and analysis of nerve conduction velocity (NCV) are common and reliable methods to evaluate the electrophysiological properties of a muscle in order to determine the degree of peripheral nerve injury and recovery [4,5]. Our preliminary pathological morphology and molecular biology studies [6] indicated that electroacupuncture (EA) at the ST36 and Ashi acupoints could promote regeneration of the rabbit gastrocnemius (GM) by improving microcirculation perfusion, promoting the recovery of myofiber structures, and inhibiting excessive fibrosis. However, the effects of EA on the recovery of GM electrophysiological properties after contusion are not yet clear.

Therefore, in this study, we aimed to determine the effects of EA treatment on the electrophysiological properties of the GM after contusion by surface EMG and measurement of the NCV of the GM and GM branch. These analyses provided experimental evidence with which to evaluate the functional recovery of the GM after contusion and allowed us to explore the mechanisms mediating the effects of EA, yielding important insights into the potential clinical applications of EA.

## Methods

### Animal model and experimental groups

Forty-five New Zealand rabbits were randomly divided into three groups: the normal group (n = 15), the contusion group with no treatment (n = 15, contusion group), and the EA-treated experimental group (n = 15, EA group). All of the animals were maintained in temperature (23 ± 1°C)-controlled and humidity (50% ± 5%)-controlled rooms under a 12-h light/dark cycle and provided water and food ad libitum. All of the experimental procedures were approved by the Ethical Committee of the Academy of Medical Sciences and were conducted in accordance with internationally accepted principles for laboratory animal use and care.

**Figure 1:**
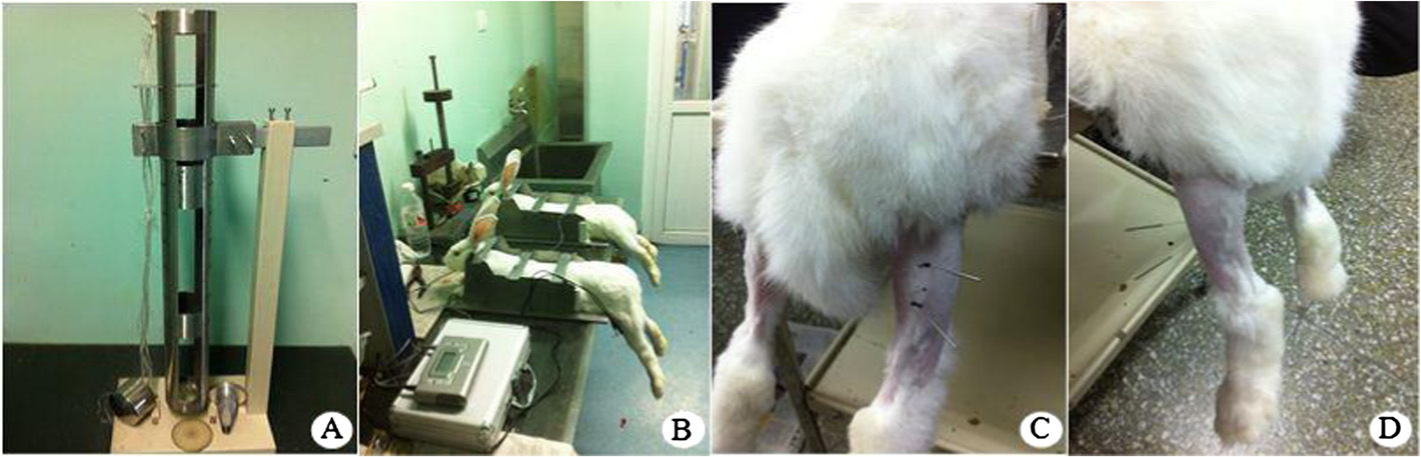
**The animal model of GM contusion and EA treatment. (A)** An acute GM contusion model device. **(B)** The fixed position of the rabbits. **(C)** EA at the Ashi acupoints of the injured side. The acupoints were located 10 mm from the proximal (anode) and distal (cathode) ends of the contusion midpoint. **(D)** EA at the ST36 acupoint of the normal side. The main needle was inserted into a standard acupoint area, and the auxiliary needle was placed 5 mm away from the main needle.

Rabbits (male or female, weighing 2.0 ± 0.2 kg each) were administered intravenous anesthesia through the marginal vein of the ear with 3% pentobarbital sodium (30 mg/kg of body weight). The animals were positioned with their right sides fixed to the experimental table. The hind limb was positioned by extending the knee and dorsiflexing the ankle to 90° to fully display the GM. Under effective anesthesia, contusion of the GM was performed using a crushing machine with the drop-mass technique and an energy of 9.555 J (Figure 1A). The injured area was approximately 1 cm^2^, located 80 mm from the rear edge of the calcaneus. While establishing this acute severe GM contusion model, which is now commonly accepted [7,8], we confirmed that the skin was intact and that there were no fractures to the tibia or fibula. Analyses were performed for five animals in each group at 7, 14, and 28 days after contusion.

### Treatments

To keep the lesions of the GM dry and avoid infection, 25 g/L Entoiodine was applied topically once per day. Beginning at 24 h after contusion, EA was administered for 15 min every other day with 0.4 mA (2 Hz). After routine disinfection, the main needle (anode; diameter: 0.25 mm, length: 25 mm; Zhongyan Taihe Medical Instruments Co. Ltd., Beijing, China) was inserted into the ST36 acupoint on the normal side (according to World Health Organization standards) to 10 mm. The auxiliary needle (cathode) was placed 5 mm from the main needle. Ashi acupoints were located 10 mm from the proximal end (anode) and distal end (cathode) of the contusion midpoint and were handled by the needle, linking the ST36 acupoint to the other acupoint areas. When all of the needles had been placed, all of the electrodes were stimulated synchronously in an identical manner using Han’s acupoint nerve stimulator (Han’s 200E; Nanjing Jisheng Medical Co. Ltd., Jiangsu, China). The rabbits in the contusion group were allowed to recover naturally from the injury, which, along with the normal group, received mock EA treatments (i.e., animals were placed into the same position at the same time after contusion as animals in the EA group, but without insertion of needles or EA treatment). All treatments were carried out without anesthesia (Figure 1B–D).

### EMG

Surface EMG of the GM was performed by the same personnel at a controlled temperature (22 ± 1°C). The experimental instrument used was a BIOPAC EMG instrument (BIO PAC MP150 16 conductive physiological recorder; BIO PAC Co. Ltd., Aero Camino Goleta, CA, USA), and data were recorded using AcqKnowledge software 4.1 (Upward Teksystems Co. Ltd., Hong Kong, China). Rabbits were administered intravenous anesthesia through the marginal vein of the ear with 3% pentobarbital sodium (30 mg/kg of body weight). For skin preparation, the hair of the hind limb was removed, and the skin was cleaned. A conductive gel (Conductive Paste, GT 20, GREENTEK Corporation, Wuhan, China) was then applied. Stimulation of the GM branch of the tibial nerve was accomplished via a 10-mm incision in the popliteal fossa, and the nerve was placed on a silver hook stimulating electrode. The anode end of the recording electrode was glued to the midpoint surface of the GM, and the cathode end was glued to the subcutaneous tissue of the ankle [9,10]. The stimulation parameters were set at a 0.12 mA direct current, rectangular square wave, and 1000 Hz stimulating frequency, and the EMG amplitude (mv) was then obtained.

### NCV Detection

To detect the conduction velocity of the motor nerve that innervates the GM, we used the software described above and selected the nerve conduction channel. The anode end of the stimulating electrode was placed beside the sacrococcygeal region, and the cathode end was placed in the subcutaneous tissue of the GM. The anode end of recording electrode 1 was placed via a 10 mm incision in the popliteal fossa, in contact with the nerve, and the cathode was glued to the skin 10 mm from the anode. The anode of recording electrode 2 was placed on the midpoint surface of the GM, and the cathode end was glued to the subcutaneous tissue of the ankle. The stimulation parameters were set at a 0.6 mA direct current and 1000 Hz stimulating frequency. Next, the nerve conduction time (ms) from recording electrode 1 to 2 and the distance (cm) between anode ends of the recording electrodes were measured. Finally, the NCV was calculated using the following formula:

$$ \mathrm{N}\mathrm{C}\mathrm{V}\left(m/s\right)=\frac{\mathrm{distance}\kern0.5em \mathrm{between}\kern0.5em \mathrm{two}\kern0.5em \mathrm{recording}\kern0.5em \mathrm{points}\left(\mathrm{cm}\right)\times 1{0}^{\hbox{--} 2}}{\mathrm{segment}\kern0.5em \mathrm{nerve}\kern0.5em \mathrm{conduction}\kern0.5em \mathrm{time}\left(\mathrm{ms}\right)\times 1{0}^{\hbox{--} 3}} $$

### Immunohistochemistry

Rabbits were sacrificed, and the GM tissue was collected. The damaged area of the tissue was cut into two equal parts. The first was fixed in 4% formalin for 3 days and then processed via gradient alcohol dehydration and paraffin embedding. The GM was cut into 5-μm sections perpendicular to the direction of the myofibers, and gradient alcohol dewaxing and hydration were then performed. The sections were placed in 3% H_2_O_2_ at room temperature for 10 min to block endogenous peroxidase activity and were then incubated with anti-Agrin (1:50) rabbit polyclonal antibodies (Abcam Co. Ltd., UK) overnight at 4°C. After washing, the sections were incubated at 37°C for 30 min with horseradish peroxidase (HRP)-conjugated anti-rabbit IgG (Wuhan Boster Biological Engineering Co. Ltd.). Next, 3′-3′diaminobenzidine (DAB; Wuhan Boster Biological Engineering Co. Ltd.) staining was applied for 10 min at room temperature. The appearance of a brown stain in the cytoplasm and nucleus signaled a positive result. When all of the staining was completed for each sample, five sections were selected (centered on the damage area, taking upper left, lower left, upper right, lower right, and intermediate sections) from immunohistochemical sections (visualized at 400×) for image acquisition. The mean optical density (MOD) value of each sample was calculated as the average of five sections using Image-Pro Plus Image analysis software (IPP, Version 6.0, Media Cybernetics, USA). Blind observations of prepared sections were made with light microscopy (ZEISS Scope. AI, Carl Zeiss, Germany).

The remaining tissue portions were wrapped in foil, frozen in liquid nitrogen, and stored at −80°C. Samples were stained using GENMED frozen section acetylcholinesterase (AchE) activity staining kits (GENMED Scientifics Inc., USA). The frozen sections were washed with a cleaning fluid, and the working solution was then applied in a drop-wise manner. Sections were incubated at 37°C for 1 h in the dark. After washing again, the frozen sections were counterstained with a GENMED hematoxylin redyeing kit (GMS80067; GENMED Scientifics Inc.) and finally were mounted in neutral gum. Dark brown staining represented positive results. When all of the staining was completed for each sample, five sections (centered on the damage area, taking upper left, lower left, upper right, lower right, and intermediate sections) were selected (visualized at 200× magnification) for image acquisition. As mentioned above, the MOD value of each sample was calculated using the averages of five sections for each sample. Blind observations of prepared sections were made with light microscopy (BX51 Scope, Olympus, Japan).

### Statistical analysis

Data were analyzed with SPSS 13.0 statistical software (SPSS Inc., Chicago, IL, USA), and results are expressed as the means ± standard deviations (SDs). Data were normally distributed and analyzed with one-way analysis of variance. Comparisons between groups were performed using the two-tailed least significant difference (LSD) method. Differences with *P* values of less than 0.05 were considered statistically significant.

## Results

### EMG amplitudes

Surface EMG represents the motor unit potential of the GM, providing information about the integrity of innervation and the condition of neuromuscular junctions and myofibers [5]. The amplitude is a reliable index used to reflect the compound action potential amplitude level; the greater the amplitude, the greater the number of excited myofibers. Thus, detection of the EMG amplitude can be used to evaluate GM regeneration.

**Figure 2:**
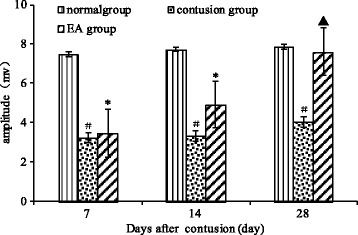
**Comparison of EMG amplitudes at different time points after contusion.** The EMG amplitude was measured in all groups at the indicated times. Contusion versus normal, ^#^ *P* < 0.05; EA versus contusion, ^▲^ *P* < 0.05; EA versus normal, ^*^ *P* < 0.05 (n = 5).

We found that the EMG amplitude in the contusion group was always significantly lower than that in the normal group (*F* =11.04, *P* = 0.01; *F* =20.72, *P* =0.02, and *F* =13.20, *P* = 0.04,). On days 7 and 14, there were no significant differences observed between the EA and contusion groups; however, on day 28, the amplitude of the EMG was significantly higher in the EA group than in the contusion group (*F* =7.42, *P* =0.03). Moreover, the reduction in EMG amplitude was greater in the EA group than in the normal group on days 7 and 14, but was similar to that of the normal group on day 28 (*F* =0.04, *P* =0.84; Figure 2).

### NCVs

**Figure 3:**
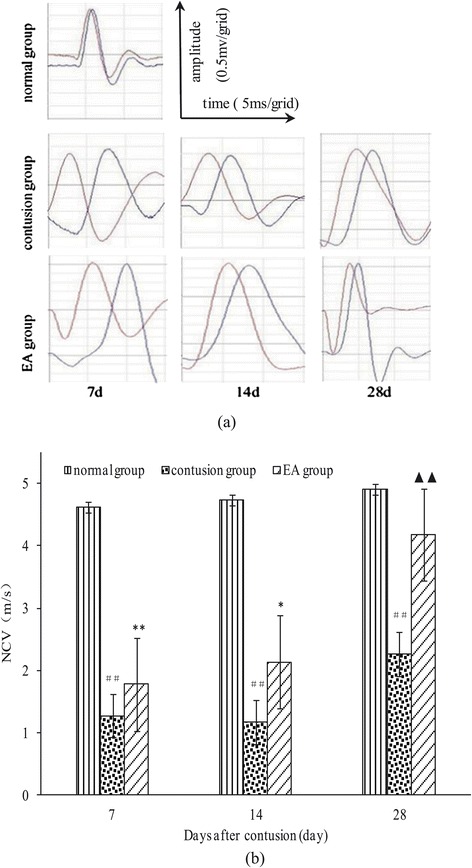
**Comparison of NCVs at different time points after contusion. (a)** NCVs under the condition of fixed transmission distance. The red wave represents electrode 1, and the blue wave represents electrode 2). **(b)** NCVs at days 7, 14, and 28 in the EA, contusion, and control groups. Contusion versus normal, ^##^ *P* < 0.01; EA versus contusion, ^▲▲^ *P* < 0.01; EA versus normal, ^*^ *P* < 0.05, ^**^ *P* < 0.01 (n = 5).

NCV can be used to detect nerve conduction in motor neurons that innervate the GM, reflecting the integrity of neurons and myelin [11]. GM injury is accompanied by nerve injury; therefore, when myofiber regeneration occurs, neurons also gradually regenerate and make contact with myofibers. Additionally, NCV acceleration occurs. In our analysis, as the time after contusion increased, the NCV of the contusion group remained slower than that of the normal group (*F* =93.01, *P* < 0.01; *F* =14.08, *P* =0.006, and *F* =22.37, *P* =0.001,). The nerve conduction time (ms) gradually shortened (i.e., accelerated) in the EA and contusion groups, while NCV gradually increased. Furthermore, the NCV of the EA group was significantly faster than that of the contusion group on day 28 (*F* =17.20, *P* <0.01), but slower than that of the normal group, although the difference was not statistically significant (*F* =2.54, *P* =0.15; Figure 3a and b).

### Expression of AchE following contusion

**Figure 4:**
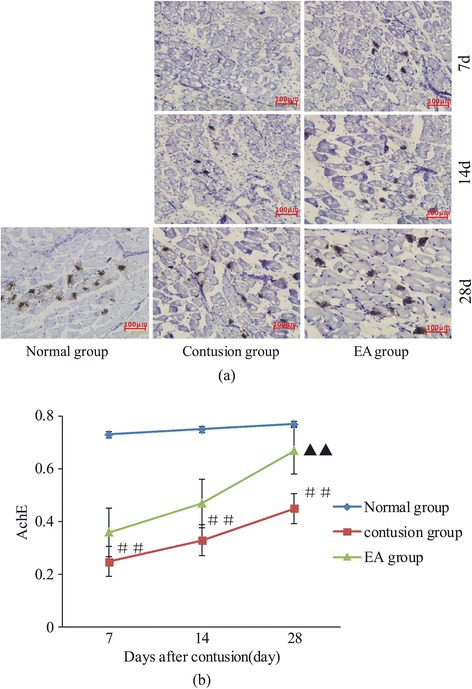
**Expression of AchE in the NMJ at different times after contusion. (a)** AchE GENMED frozen section staining at the NMJ (200×) at different time points after contusion. **(b)** Comparison of AchE expression levels at different times after contusion. Contusion versus normal, ^##^ *P* < 0.01; EA versus contusion, ^▲▲^ *P* < 0.01 (n = 5).

The expression and concentration of AchE at the postsynaptic membrane signifies the integrity of the structure and function of the NMJ. In the normal group, AchE expression was observed at the NMJ and steadily accumulated in the motor endplate area. Through staining, dark brown, tiny particles were observed; these particles were oval or round and clear in the coronal section. After the GM injury, the NMJ was broken. On day 7, a small amount of AchE was detected in the NJM of samples from the contusion group, and with time, the NMJ structure was gradually restored, with gradual accumulation of AchE expression at the postsynaptic membrane. On days 7, 14, and 28, the MOD value of AchE in the contusion group was markedly lower than that in the normal group (*F* =21.11, *P* = 0.02; *F* =28.16, *P* = 0.01 and *F* =14.07, *P* = 0.06). In contrast, AchE expression in the EA group was obviously stronger than that in the contusion group. The differences in MOD values between the EA and contusion groups were still significantly different on day 28 (*F* =6.39, *P* =0.04). However, by day 28, no differences in AchE expression in the motor endplate area were observed between the EA and normal groups (*F* =4.68, *P* =0.06; Figure 4a and b).

### Agrin expression following contusion

**Figure 5:**
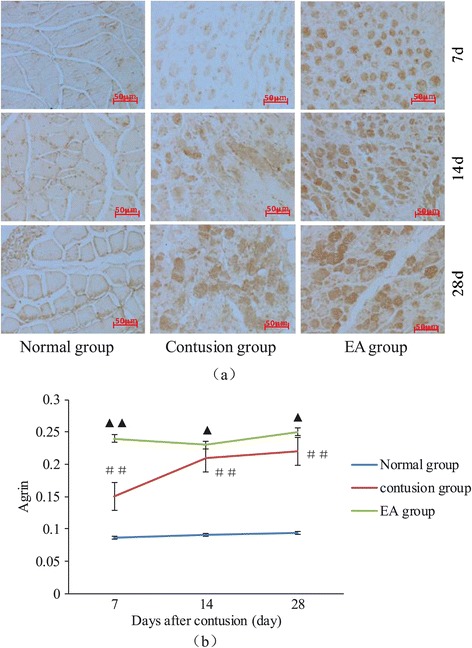
**Expression of Agrin in the NMJ at different times after contusion. (a)** DAB staining of Agrin in the NMJ on days 7, 14, and 28 after contusion (400×). **(b)** Comparison of the expression levels of Agrin on days 7, 14, and 28. Contusion versus normal, ^##^ *P* < 0.01; EA versus contusion, ^▲▲^ *P* < 0.01, ^▲^ *P* < 0.05 (n = 5).

Agrin is present in the functional reconstruction phase of the NMJ, particularly in the postsynaptic membrane, where it plays a key role. Under physiological conditions, a small amount of Agrin was expressed at the motor endplate area; however, Agrin expression was absent immediately after motor endplate area injury. Over time, Agrin expression in the contusion group gradually increased, and the MOD value was markedly higher than that in the normal group (*F* =178.38; *F* =326.38, and *F* =294.72, *P* < 0.01). In contrast, recovery of Agrin expression was substantially faster in the EA group than in the contusion group, and the MOD value was also obviously higher than that the contusion group (*F* =405.7, *P* <0.01; *F* =10.64, *P* =0.011, and *F* =20.44, *P* = 0.012; Figure 5a and b).

## Discussion

Researchers have shown that EA can significantly promote tissue repair in such contexts as myocardial infarction, nerve regeneration, and wound healing [12-15]. Moreover, EA can also accelerate muscle regeneration and improve the quality of repair after skeletal muscle injury [6]. Our previous studies showed that EA treatment reduces the expression of both GDF-8 and p-Smad2/3, decreases the formation of fibrotic tissue in injured muscle, increases the expression of myosin heavy chain (MHC), and enhances the capillary density at injured sites, thereby improving muscle fiber regeneration [6]. However, whether EA can promote the functional recovery of skeletal muscle is still unclear. Thus, in this study, we conducted a series of experiments to determine the effects of EA on recovery of the electrophysiological properties of rabbit GM after contusion. Our data showed that EA enhanced recovery after contusion compared to untreated conditions, supporting the potential use of EA in clinical applications for muscle repair.

### EA treatment promoted motor unit potential and nerve conduction recovery, improving the electrophysiological properties of skeletal muscle

A motor unit is a functional unit of contracting skeletal muscle that contains a motor neuron and all dominating myofibers, each with its own rich blood supply. EA promotes the recovery of neurons, myofibers, and angiogenesis of injured motor units [16]. In our study, EA treatment resulted in gradual restoration of the structure of the motor unit, and the motor unit was eventually recovered to nearly normal in terms of structure and function. EMG was used to detect the sum of the potential of all of the motor neurons in the process of a muscle contraction. The amplitude of the EMG readout is proportional to the number of excited myofibers, thus reflecting the function of motor units [17]. Moreover, changes in the EMG not only reflect the repair process of the GM after injury but also the number and quality of myofibers repaired and regenerated after injury. In a previous study, injection of injured skeletal muscle with insulin-like growth factor-I (IGF-1) accelerated recovery of the EMG amplitude, which returned to normal by days 28–35 after injury (as compared to the lack of full recovery after 56 days in the contusion group) [18]. In our study, on day 7, the EMG amplitudes in the EA and contusion groups were low; however, by day 14, the amplitudes had increased, albeit not to the level of the normal group. Thus, it is possible that the injured myofiber structure was not adequately recovery, and the injured muscle had not yet obtained normal contact with the nerve. Importantly, by day 28, the EMG amplitude in the EA group had recovered nearly to that in the normal group, indicating that the EA treatment had a beneficial, therapeutic effect regardless of the number of motor units or their restored quality, achieving better outcomes than in the untreated contusion group.

Skeletal muscle injury caused by contusion can damage the NMJ and nerves and cause secondary damage to nerve fibers due to obstruction of blood circulation [19]. Regenerated myofibers can form functional motor units only by being innervated [20,21], and motor neuron regeneration and NMJ reconstruction following muscle injury can be evaluated by measurement of NCV. In our study, following EA treatment, the NCV gradually increased, reaching near normal velocity by day 28. Therefore, our data showed that EA could promote GM fiber regeneration and restoration of nerves innervating the GM and motor units, indicating that EA had multiple effects. Indeed, in previous studies, EA treatment has been shown to promote the continuous growth of glial cells (i.e., Schwann cells) [22] and enhance the synthesis of neurotrophic factors, such as nerve growth factor [23], ciliary neurotrophic factor [24], NT-3/NT-4 [25], and IGF-1 [26], which play important roles in the regeneration of injured nerves [27]. Huang et al. [13] showed that EA increased the number and diameter of axons, thickened the myelin sheath, and increased the number of motor neurons during the delayed recovery of the sciatic nerve, thereby promoting the functional recovery and regeneration of nerves. Thus, taken together with our current data, these results suggest that EA may have various applications in the recovery of muscle structure and function (e.g., nerve regeneration).

### EA treatment facilitated recovery of the electrophysiological properties of the GM and promoted the restoration of NMJ function

The structure and function of the NMJ, which acts as the link between nerves and muscles, play key roles in the normal function of the skeletal muscle. Newly formed (i.e., recovering) NMJs have been shown to have the capacity for endplate potentials; however, contraction of myofibers is not achieved [28], that is, the NMJ did not perform its original function, despite the completion of nerve regeneration and sufficient contact between myofibers and neurons. Moreover, original reconstructed NMJ has been shown to exhibit incomplete function, and the reconstruction of NMJ function (i.e., the impulse) still requires the auxiliary function of nerve and muscle signaling molecules [29-32].

In our study, on day 28 after EA treatment, recovery of the EMG amplitude and NCV was achieved, suggesting that EA was an effective intervention for NMJ signaling molecules. The neurotransmitter acetylcholine (Ach) was released into the synaptic cleft, part of which was decomposed by AchE (which was present in the synaptic cleft), part which was absorbed by the presynaptic membrane to be reused, and part of which combined with the Ach receptor (AchR) at the postsynaptic membrane to create effective nerve impulses. The density of AchR at the postsynaptic membrane directly influences the current intensity at the postsynaptic membrane and neurotransmitter release at the NMJ to cause effective nerve impulses [33]. Thus, it is possible that treatment with EA promoted the recovery of the electrophysiological properties of the GM in addition to enhancing nerve repair and myofiber regeneration. Recovery of NMJ function can also be achieved by increased synthesis of Ach, AchE, and AchR, which accumulate steadily at the postsynaptic membrane [32].

AchE is a key enzyme involved in cholinergic nerve conduction and is primarily expressed in neurons and the NMJ. Its physiological function is to quickly hydrolyze the neurotransmitter Ach, ultimately causing Ach to activate cholinergic receptors while maintaining the sensitivity of nerve impulses. Therefore, the presence of AchE at the postsynaptic membrane reflects the recovery of NMJ function [32]. In our study, we examined AchE as a tissue marker to verify the role of EA in facilitating accumulation of AchE at the postsynaptic membrane. Over time, AchE expression gradually increased in the motor endplate in the EA group, as demonstrated by gradually stronger staining, reaching nearly normal levels by day 28. Interestingly, AchE also accumulated in the newly formed (i.e., recovering) NMJ. Thus, our data showed that EA promoted the return of function to the NMJ, consistent with the accumulation of AchE expression in the NMJ.

Agrin also plays a key role in the physiological processes of the NMJ, especially the postsynaptic membrane, which is involved in the formation, differentiation, maturation, and consistency of the NMJ [34]. The Agrin/muscle-specific receptor tyrosine kinase (MuSK)/Rapsyn signaling pathway has been shown to be critical for postsynaptic gathering and decomposition of the AchR [32]. Within this pathway, Agrin activates postsynaptic MuSK by interacting with Lrp4 receptors. Phosphorylated MuSK then interacts with dok-7, effectively increasing AchR postsynaptic gathering, promoting the interaction between AChR and Rapsyn in the newly formed NMJ [35,36], stabilizing the AchR, and slowing the decomposition of the AChR. In our study, under physiological conditions, a small amount of Agrin was expressed at the motor endplate area; however, Agrin expression was absent immediately after injury. After EA treatment, Agrin expressed was recovered quickly, with obviously higher expression in the EA group than in the contusion group. Thus, we speculate that EA could promote AChR clustering and stabilization in the postsynaptic membrane by upregulating the expression of the Agrin, thereby contributing to the recovery of NMJ function. Further studies are required to determine the specific mechanisms mediating these effects.

## Conclusion

In conclusion, these results suggested that EA promoted the recovery of the electrophysiological properties of skeletal muscle, including EMG amplitude and NCV, which are closely associated with regeneration of skeletal muscle, functional repair of nerves, and NMJ reconstruction. We also found that EA promoted functional recovery of the NMJ by promoting AchE and Agrin expression at the postsynaptic membrane. Further studies are required to determine the specific mechanisms mediating these effects.

## Abbreviations

EA: Electroacupuncture
ST36: Zusanli
GM: Gastrocnemius
EMG: Electromyography
NCV: Gastrocnemius branch of the tibial nerve conduction velocity
MOD: Mean optical density
AchE: Acetylcholinesterase
NMJ: Neuromuscular junction
MuSK: Muscle-specific receptor tyrosine kinase
